# The prevalence and impact of comorbidities on patients with axial spondyloarthritis: results from a nationwide population-based study

**DOI:** 10.1186/s13075-020-02301-0

**Published:** 2020-09-10

**Authors:** Imke Redeker, Johanna Callhoff, Falk Hoffmann, Ursula Marschall, Hildrun Haibel, Joachim Sieper, Angela Zink, Denis Poddubnyy

**Affiliations:** 1 Epidemiology Unit, German Rheumatism Research Centre, Charitéplatz 1, 10117 Berlin, Germany; 2 Department of Health Services Research, Carl von Ossietzky University, Oldenburg, Germany; 3 BARMER Institute for Health Systems Research, BARMER Statutory Health Insurance, Wuppertal, Germany; 4 Department of Gastroenterology, Infectiology and Rheumatology, Charité – Universitätsmedizin Berlin, Berlin, Germany; 5 Department of Rheumatology and Clinical Immunology, Charité – Universitätsmedizin Berlin, Berlin, Germany

**Keywords:** Axial spondyloarthritis, Comorbidity, Multimorbidity, Disease activity, Functional status

## Abstract

**Background:**

In contrast to other chronic rheumatic musculoskeletal diseases such as rheumatoid arthritis, comorbidities in axial spondyloarthritis (axSpA) and their impact on disease outcomes are less well studied. The aim of this study was to investigate the prevalence of comorbidities and their association with disease activity and functional impairment in a large population-based cohort of patients with axSpA.

**Methods:**

A random sample of patients with axSpA, stratified by age and sex, was drawn from health insurance data. Patients in the sample received a survey on demographic, socioeconomic, and disease-related parameters. Comorbidities were defined using the Elixhauser coding algorithms excluding rheumatoid arthritis/collagen vascular diseases and including osteoporosis and fibromyalgia, resulting in a set of 32 comorbidities. The prevalence of comorbidities in the axSpA patients and their pharmacological treatment were examined. Multivariable linear regression models were calculated to determine the association of comorbidities with disease activity and functional status.

**Results:**

A total of 1776 axSpA patients were included in the analyses (response, 47%; mean age, 56 years; 46% female). The most prevalent comorbidities were hypertension, depression, and chronic pulmonary disorders. The number of comorbidities was significantly associated with both the BASDAI and BASFI: *β* (95% CI) = 0.17 (0.09–0.24) and 0.24 (0.15–0.32), respectively. When analysed separately, hypertension, depression, and chronic pulmonary disease were comorbidities with a significant and independent association with BASFI, while for BASDAI, such an association was found for depression and chronic pulmonary disease only.

**Conclusions:**

Comorbidities are common in axSpA patients and are associated with higher disease activity and higher levels of functional impairment. Higher disease activity and higher levels of functional impairment might be indicators of severe disease resulting in the development of comorbidities.

## Key messages

- The prevalence of comorbidities in patients with axial spondyloarthritis is high.
- The overall burden of comorbidities and disease activity/function are significantly associated in axial spondyloarthritis patients.
- Patient-tailored management strategies including identification and appropriate treatment of comorbidities are needed in axial spondyloarthritis.

## Introduction

Axial spondyloarthritis (axSpA) is a term that encompasses chronic inflammatory diseases characterised by predominant involvement of the spine and/or sacroiliac joints, including non-radiographic axSpA (nr-axSpA, i.e. axSpA without definite radiographic sacroiliitis) and radiographic axSpA (also termed ankylosing spondylitis (AS), characterised by the presence of radiographic sacroiliitis according to the modified New York criteria) [1]. The predominant symptom is chronic back pain with onset occurring in early adulthood, usually before age 45. Peripheral symptoms (peripheral arthritis, enthesitis, dactylitis) might also be present as well as extra-musculoskeletal manifestations such as inflammatory bowel disease, psoriasis, and uveitis, contributing to the total burden of axSpA [2].

In addition to those manifestations, which are directly related to axSpA, patients may also suffer from other diseases referred to as comorbidities [3]. In contrast to other chronic rheumatic musculoskeletal diseases such as rheumatoid arthritis (RA), comorbidities in axSpA and their impact on disease outcomes are less well studied.

Evidence on the prevalence of some comorbidities in SpA and their association with disease outcomes was obtained by the international Assessment in SpondyloArthritis International Society (ASAS)-COMOSPA study [4]. In particular, the authors found osteoporosis and gastrointestinal ulcer to be the most prevalent and second most prevalent comorbidities in SpA, respectively [4], and demonstrated that a higher comorbidity burden in SpA was associated with worse functional and work-related outcomes and poorer quality of life [5].

A common approach among studies (usually cohort studies and registries) of comorbidity in SpA had been to collect data on selected comorbidities only rather than to look at all comorbidities first to subsequently identify the most prevalent and relevant ones. In a recent study from the UK, comorbidities were selected based on their importance in the general UK population, and comorbidity clusters in axSpA were identified [6]. Another approach is to select comorbidities based on the Elixhauser Index, which has been developed for use in administrative claims databases and is one of the most widely used and validated indices in comorbidity research [7–10].

The objective of this population-based study was to investigate the prevalence of comorbidities and their association with disease activity and functional impairment in a large sample of patients with axSpA by using survey data linked to health insurance data obtained within the Linking Patient-Reported Outcomes with CLAIms data for health services research in Rheumatology (PROCLAIR) network [11].

## Patients and methods

### Study design

A detailed description of the study design has been reported elsewhere [12]. Briefly, a random sample, stratified by age and sex, of 5000 out of 21,892 patients with a diagnosis of axSpA [International Classification of Diseases, 10th Revision (ICD-10) code M45] in at least two quarters of the year 2014 was drawn from health insurance data in Germany. All selected patients received a survey in 2015 (a reminder was sent out to those patients who had not answered within 4 weeks) to gather information on disease-related, demographic, and socioeconomic parameters including information on disease activity, assessed using the Bath Ankylosing Spondylitis Disease Activity Index (BASDAI) [13], and functional status, assessed using the Bath Ankylosing Spondylitis Functional Index (BASFI) [14]. Psychological well-being was assessed using the 5-item WHO Well-Being Index (WHO-5) [15]. Survey data were linked to health insurance data from 2015 to gather further information on comorbidities, drug prescriptions, and physical therapy; the latter included exercise therapy, manual therapy, and massage by a physiotherapist. AxSpA-related pharmacological treatment comprised non-steroidal anti-inflammatory drugs (NSAIDs), biological disease-modifying anti-rheumatic drugs (bDMARDs), non-opioid analgesics, opioids, conventional synthetic disease-modifying anti-rheumatic drugs (csDMARDs), and steroids.

### Comorbidities and their treatment

The comorbidities included in this analysis were based on ICD-10 codes and defined using the Elixhauser coding algorithms [16], slightly modified by excluding rheumatoid arthritis/collagen vascular diseases and including osteoporosis [ICD-10 codes M80-M82] and fibromyalgia [ICD-10 code M79.7] based on previous evidence on their relevance in SpA [3]. In the resulting set of 32 comorbidities, complicated and uncomplicated forms of hypertension and diabetes are distinguished. Patients who had ICD-10 codes for both forms were assigned to the condition with complications. For comorbidities, at least one inpatient or outpatient claim had to be documented. Treatment of comorbidities was investigated using drug prescriptions in outpatient care based on the Anatomical Therapeutic Chemical (ATC) classification.

### Population-based cohort of non-axSpA patients

A sex- and age-matched cohort of patients who were continuously insured in 2013 and 2014 and had no recorded axSpA diagnosis (i.e. no recorded ICD-10 code M45) was drawn in a 1 to 5 ratio from the health insurance data. There were no further restrictions in terms of recorded diagnoses. The prevalence of comorbidities in this matched cohort of non-axSpA patients, regarded as population-based controls, was examined.

### Statistical analysis

The total number of persons who responded to the survey and gave their consent for linking survey data to health insurance data was weighted according to the sex and age group distribution of the source population of 21,892 patients with a diagnosis of axSpA. Weighted subgroup analyses were performed on those who confirmed their axSpA diagnosis. All variables obtained from survey data had a maximum of 4% missing values, except for the household income (6% missing values) and HLA-B27 status (31% missing values) variables. Missing data values were not imputed.

The prevalence and treatment of comorbidities in axSpA patients were assessed, and differences between axSpA patients with none, 1–2, 3–4, and ≥ 5 comorbid conditions were examined using descriptive statistics (means, standard errors of the mean (SEMs) and percentages). The SEM was used instead of the standard deviation to account for the stratified nature of the study design. Significant differences were assessed using one-way analyses of variance for continuous variables and using Rao-Scott chi-square tests otherwise. The latter is a version of the Pearson chi-square test, used to adjust for the stratified nature of the study design. Tests resulting in *p* values < 0.05 were considered statistically significant. In addition, the prevalence of comorbidities in the matched cohort of non-axSpA patients, who were weighted similarly to the axSpA patients, was investigated.

Univariable and multivariable linear regression models were calculated to analyse the association of [1] the number of comorbidities and [2] specific comorbidities with disease activity and functional impairment, with a focus on explanation. Variables included in the multivariable models were chosen using backward selection whereby the set of tested variables comprised the number of comorbidities (only model 1), comorbidities with prevalence > 5% taken separately (only model 2), age, sex, symptom duration, in rheumatologic care, HLA-B27, psoriasis, inflammatory bowel disease, uveitis, body mass index (BMI), lack of exercise, smoking, suffering from stress, household income, axSpA-related medication (NSAIDs, bDMARDs, non-opioid analgesics, opioids, csDMARDs, and steroids), number of pharmaceuticals (excluding axSpA-related medication), and physical therapy. Age and sex were always included in the models. A significance level of 0.05 was required for a variable to stay in the multivariable model. Parameter estimates (*β*) were calculated with 95% confidence intervals (CIs). The multivariable regression analyses were repeated with comorbidities only assumed to be present if indicative medications were prescribed.

Data analyses were performed with SAS 9.4 (SAS Institute Inc., Cary, NC) using procedures for complex survey designs.

### Patient and public involvement

A focus group was set up and actively contributed to the development of the survey design.

## Results

A total of 4471 patients with an axSpA diagnosis (5000 of the original sample minus those who died or changed their insurance) received the survey and 2118 patients responded (47%). Of those, a total of 2082 patients gave their consent for linking survey data to health insurance data, among whom 1776 patients confirmed their axSpA diagnosis (85%) and were therefore included in the analysis. The mean age was 56 years, and 46% were female. Both characteristics were comparable between those patients who responded to the survey and those who did not respond. The mean number of comorbidities was 2.5, and the mean number of pharmaceuticals was 7.2. While the first characteristic was comparable between responders and non-responders, the latter was increased among responders.

### Patients’ characteristics stratified by the number of comorbidities

In the entire axSpA group, 17% of the patients had no comorbid conditions, 41% had 1–2 comorbid conditions, 25% had 3–4 comorbid conditions and 17% had 5 or more comorbid conditions. The main demographic, disease-related, and socioeconomic characteristics are presented in Table 1 for the total group of 1776 axSpA patients and stratified by the number of comorbidities. AxSpA patients with no additional comorbid conditions were on average 20 years younger, with shorter disease duration and with shorter diagnostic delay than axSpA patients with 5 or more comorbidities. The prevalence of psoriasis was significantly higher in patients with 5 or more comorbidities (19%) than in patients with no comorbidities (9%), whereas uveitis and inflammatory bowel disease were equally prevalent in all groups.

**Table 1:** Characteristics of patients with axial spondyloarthritis (*N* = 1776) stratified by the number of comorbidities

	Total, ***N*** = 1776 (100%)	Number of comorbidities*				***P*** value
		0, ***N*** = 315 (17%)	1–2, ***N*** = 741 (41%)	3–4, ***N*** = 439 (25%)	≥ 5, ***N*** = 281 (17%)	
Female sex (%)	46.4	38.6	49.2	49.1	43.0	**0.0046**
Age, years (mean ± SEM)	56.1 ± 0.1	45.4 ± 0.6	53.8 ± 0.4	60.3 ± 0.5	65.9 ± 0.5	**< 0.0001**
Symptom duration, years (mean ± SEM)	25.3 ± 0.3	18.9 ± 0.7	24.3 ± 0.5	28.2 ± 0.7	30.2 ± 0.9	**< 0.0001**
Duration since diagnosis, years (mean ± SEM)	19.5 ± 0.3	14.2 ± 0.6	18.4 ± 0.5	22.0 ± 0.6	24.1 ± 0.9	**< 0.0001**
In rheumatologic care (%)	45.6	51.6	44.1	44.6	44.5	0.1578
HLA-B27-positive (%)	86.1	87.8	88.7	83.2	79.8	**0.0211**
Psoriasis (ever, %)	15.0	8.5	15.7	15.4	19.0	**0.0047**
IBD (ever, %)	8.8	10.8	6.6	9.8	10.8	0.0618
Uveitis (ever, %)	27.3	24.7	27.7	29.1	26.6	0.6134
BASDAI, 0–10 (mean ± SEM)	4.5 ± 0.0	3.7 ± 0.1	4.3 ± 0.1	4.8 ± 0.1	5.2 ± 0.1	**< 0.0001**
BASFI, 0–10 (mean ± SEM)	4.1 ± 0.1	2.7 ± 0.1	3.7 ± 0.1	4.7 ± 0.1	5.6 ± 0.1	**< 0.0001**
Body mass index, kg/m^2^ (mean ± SEM)	26.9 ± 0.1	25.2 ± 0.2	26.2 ± 0.2	28.2 ± 0.2	28.8 ± 0.3	**< 0.0001**
WHO-5 Well-being Index, 0–100 (mean ± SEM)	44.7 ± 0.5	47.4 ± 1.2	45.5 ± 0.8	44.5 ± 1.1	40.2 ± 1.3	**0.0005**
Smoking (current, %)	18.6	22.1	21.0	15.3	13.9	**0.0076**
Suffering from stress (%)	39.3	46.2	43.3	37.3	25.2	**< 0.0001**
Lack of exercise (%)	24.3	20.5	20.8	28.8	30.8	**0.0004**
Full-time employment (%)	31.1	54.3	38.5	19.8	6.2	**< 0.0001**
Household income, € (%)						
< 1500	26.2	18.1	25.1	24.1	40.2	**< 0.0001**
1500–3200	55.9	54.3	54.6	60.0	54.2	
> 3200	17.9	27.6	20.4	15.8	5.6	
Pharmacotherapy (%)	77.9	75.5	75.8	79.2	83.4	**0.0452**
NSAIDs	59.0	56.7	60.5	60.5	55.3	0.3413
Non-opioid analgesics	22.5	16.7	17.8	28.1	31.5	**< 0.0001**
Opioids	16.0	8.9	12.3	17.3	30.5	**< 0.0001**
bDMARDs	16.9	26.0	19.7	11.8	8.6	**< 0.0001**
csDMARDs	13.6	13.5	14.5	13.1	12.2	0.7783
Steroids	19.7	12.1	17.9	23.7	25.8	**< 0.0001**
Physical therapy (%)	52.1	46.6	45.5	57.9	65.2	**< 0.0001**
Number of pharmaceuticals (mean ± SEM)	7.2 ± 0.1	3.7 ± 0.1	5.9 ± 0.1	8.2 ± 0.2	11.9 ± 0.3	**< 0.0001**
Number of pharmaceuticals** (mean ± SEM)	5.6 ± 0.1	2.5 ± 0.1	4.3 ± 0.1	6.4 ± 0.2	9.9 ± 0.3	**< 0.0001**

*Defined by the Elixhauser coding algorithms excluding rheumatoid arthritis/collagen vascular diseases and including osteoporosis and fibromyalgia. **excluding axSpA-related medication. *P* values < 0.05 are shown in bold

*AxSpA* axial spondyloarthritis, *BASDAI* Bath Ankylosing Spondylitis Disease Activity Index, *BASFI* Bath Ankylosing Spondylitis Functional Index, *bDMARDs* biological disease-modifying anti-rheumatic drugs, *csDMARDs* conventional synthetic disease-modifying anti-rheumatic drugs, *IBD* inflammatory bowel disease, *NSAIDs* non-steroidal anti-inflammatory drugs, *SEM* standard error of the mean

**Fig. 1:**
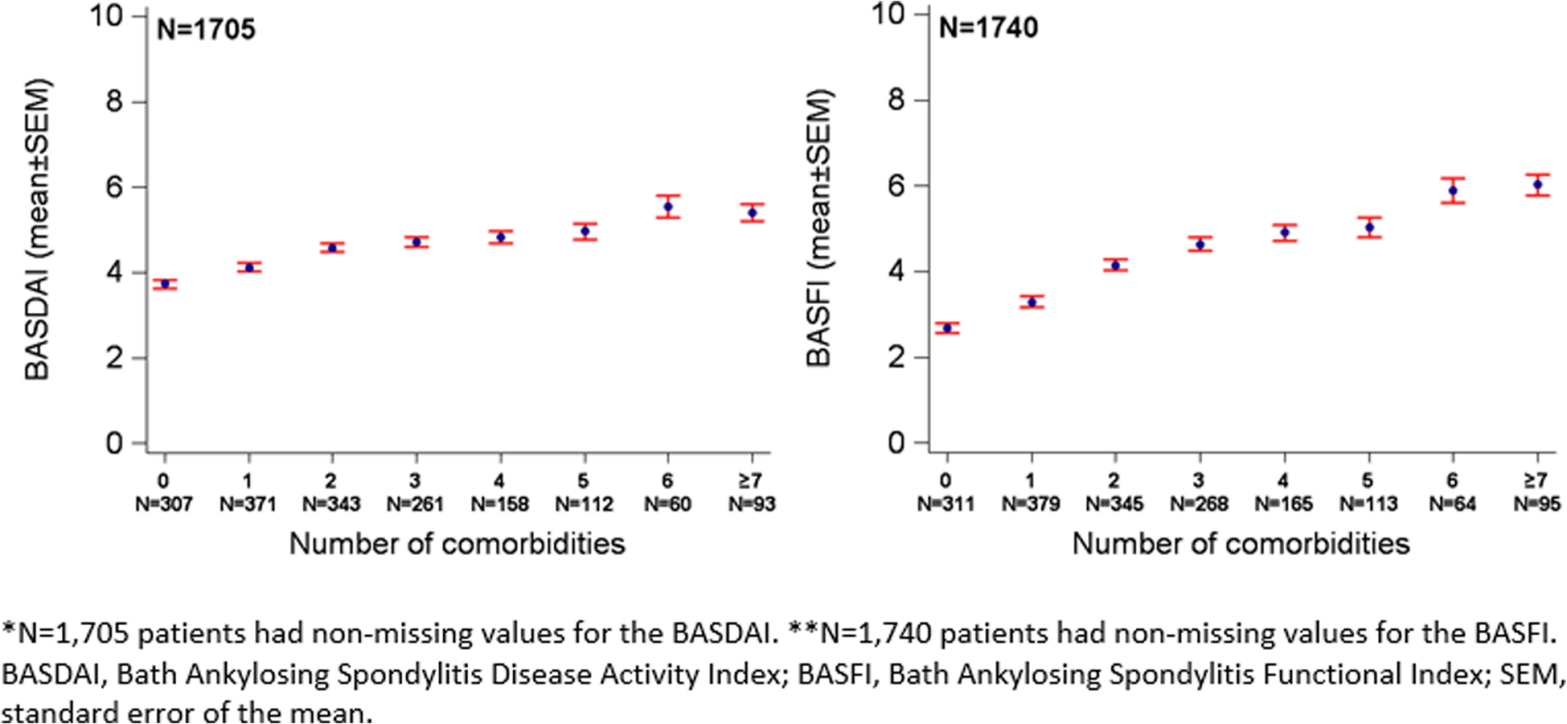
Disease activity (BASDAI*) and functional impairment (BASFI**) by number of comorbidities in axial spondyloarthritis patients

Patients with no comorbidities were more often in rheumatologic care than patients with 5 or more comorbidities (52% vs 45%). Disease activity (BASDAI) and functional impairment (BASFI) worsened with an increasing number of comorbidities (Fig. 1). The mean BASDAI/BASFI increased from 3.7/2.7 for patients with no comorbidities to 5.4/6.0 for patients with 7 or more comorbidities.

Significant differences between the groups were observed in socioeconomic and lifestyle characteristics. Patients with 5 or more comorbidities more often had a lower household income and were less often employed full-time than axSpA-only patients. They also had a higher BMI and lower psychological well-being (WHO-5). Patients with no comorbid conditions were more often smoker (22%) and suffered more often from stress (46%) than patients with 5 or more comorbidities (14% and 25%, respectively). A lack of exercise was reported by 21% of axSpA patients without comorbidities compared to 31% of axSpA patients with 5 or more comorbidities.

The prescription of NSAIDs and csDMARDs was similar between the groups, whereas significant differences were found for treatment with biological agents, opioids, non-opioid analgesics, and steroids. Patients with no comorbid conditions received biological agents more often than patients with 5 or more comorbidities (26% vs 9%), but they received opioids, non-opioid analgesics, and steroids less often. Physical therapy was more often prescribed for patients with 5 or more comorbidities than it was for axSpA-only patients (65% vs 47%). The mean number of pharmaceuticals increased from 3.7 for patients with no comorbid conditions to 11.9 for patients with 5 or more comorbidities and from 2.5 to 9.9 if axSpA-related treatment was excluded.

### Prevalence of comorbidities and their treatment

**Fig. 2:**
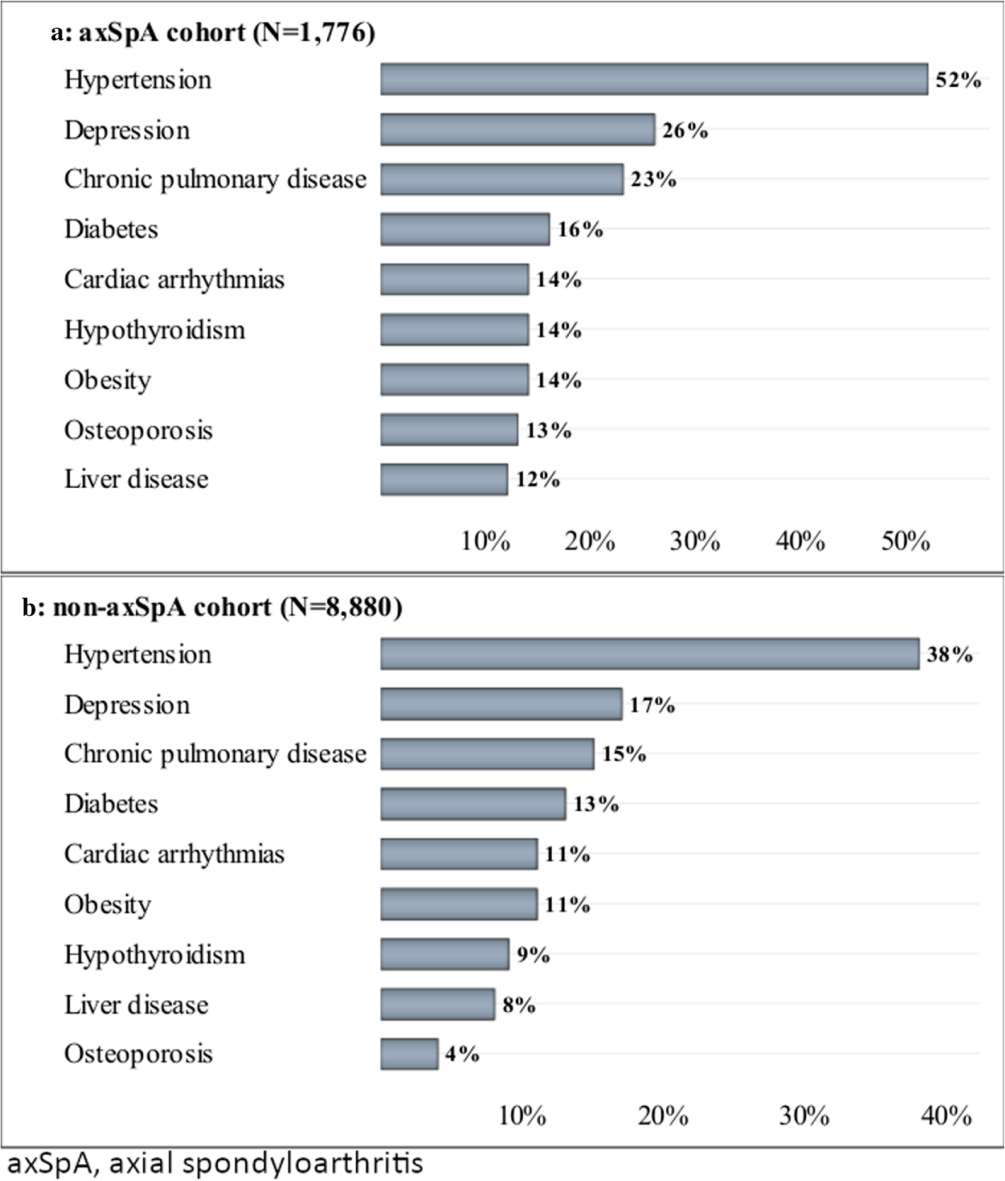
Most prevalent comorbidities in axSpA patients (**a**) in relation to non-axSpA patients (**b**)

The prevalence of investigated comorbidities is outlined in Table 2. The most prevalent comorbid condition was hypertension (52%), followed by depression (26%); chronic pulmonary disease (23%), where asthma was present in 10% and chronic obstructive pulmonary disease in 9% of the axSpA patients; and diabetes (16%). Figure 2 illustrates the most prevalent comorbidities in axSpA patients (A) in relation to the matched cohort of non-axSpA patients (B).

**Table 2:** Comorbidities defined by Elixhauser coding algorithms* in axial spondyloarthritis patients (*N* = 1776)

Comorbidity	Prevalence (%)
Hypertension	51.6
Uncomplicated	44.4
Complicated	7.2
Depression	25.6
Chronic pulmonary disease	23.4
Diabetes	16.0
Complicated	8.7
Uncomplicated	7.3
Cardiac arrhythmias	14.2
Obesity	14.2
Hypothyroidism	13.6
Osteoporosis	13.0
Liver disease	11.7
Peripheral vascular disorders	8.6
Solid tumour without metastasis	8.3
Valvular disease	8.1
Renal failure	7.5
Congestive heart failure	5.7
Deficiency anaemia	5.6
Fibromyalgia	4.4
Other neurological disorders	3.6
Coagulopathy	2.9
Fluid and electrolyte disorders	1.8
Weight loss	1.6
Alcohol abuse	1.6
Metastatic cancer	1.4
Peptic ulcer disease excluding bleeding	1.3
Pulmonary circulation disorders	1.2
Paralysis	1.2
Drug abuse	1.1
Lymphoma	0.6
Psychoses	0.5
Blood loss anaemia	0.1
AIDS/HIV	0

*Excluding rheumatoid arthritis/collagen vascular diseases and including osteoporosis and fibromyalgia

Almost all axSpA patients with a complicated form of hypertension received pharmacological treatment (98%) and also a vast majority of patients with uncomplicated hypertension were treated (87%). Around half of the patients with depression or chronic pulmonary disease received related pharmacological treatment (Table 3). Pharmacological treatment of patients with a complicated form of diabetes was increased compared to patients with uncomplicated diabetes (77% vs 44%).

**Table 3:** Proportion of treated comorbidities in axial spondyloarthritis patients (*N* = 1776)

Comorbidity	Percent diagnosed	Percent treated
Hypertension without complications	44.4	87.1
Beta-blockers (ATC code C07)		49.8
ACE inhibitors (ATC codes C09AA, C09B)		37.5
Angiotensin-I-antagonists (ATC codes C09CA, C09D)		34.8
Diuretic drugs (ATC code C03)		22.7
Hypertension with complications	7.2	98.3
Beta-blockers (ATC code C07)		66.0
ACE inhibitors (ATC codes C09AA, C09B)		37.5
Angiotensin-I-antagonists (ATC codes C09CA, C09D)		49.2
Diuretic drugs (ATC code C03)		45.5
Depression	25.6	46.7
Anti-depressants (ATC code N06A)		46.7
Chronic pulmonary disease	23.4	46.9
LABA (ATC codes R03AC, R03AK, R03AL)		41.0
LAMA (ATC code R03BB)		9.6
Inhaled corticosteroids (ATC code R03BA)		13.2
Diabetes with complications	8.7	77.4
Insulin (ATC code A10A)		32.6
Oral hypoglycaemics (ATC code A10B)		60.1
Diabetes without complications	7.3	44.4
Insulin (ATC code A10A)		12.4
Oral hypoglycaemics (ATC code A10B)		38.6
Cardiac arrhythmias	14.2	68.5
Beta-blockers (ATC code C07)		65.9
Digitalis glycosides (ATC code C01AA)		5.7
Anti-arrhythmic drugs (ATC code C01BD)		3.8
Peripheral vascular disorders	8.7	63.0
Antiplatelet drugs (ATC code B01AC)		26.5
Heparin (−derivates) (ATC code B01AA)		12.3
Vitamin K antagonists (ATC code B01AB)		11.4
Statins (ATC code C10AA)		44.2
Valvular disease	8.1	73.7
Beta-blockers (ATC code C07)		62.0
ACE inhibitors (ATC codes C09AA, C09B)		35.0
Diuretic drugs (ATC code C03)		28.5
Congestive heart failure	5.7	92.5
Beta-blockers (ATC code C07)		69.2
ACE inhibitors (ATC codes C09AA, C09B)		40.2
Angiotensin-I-antagonists (ATC codes C09CA, C09D)		36.9
Digitalis glycosides (ATC code C01AA)		9.4
Diuretic drugs (ATC code C03)		53.0
Fibromyalgia	4.4	19.7
Duloxetine (ATC code N06AX21)		5.0
Pregabalin (ATC code N03AX16)		9.5
Gabapentin (ATC code N03AX12)		5.2

*ACE* angiotensin-converting-enzyme, *LABA* long-acting beta2 agonists, *LAMA* long-acting muscarinic antagonists

### Association of comorbidity with disease activity and functional impairment

The results from univariable and multivariable linear regression models analysing the association of the number of comorbidities and specific comorbidities with disease activity and functional impairment are outlined in Table 4. Further results from univariable linear regression models analysing the association of all comorbidities with a prevalence > 5% can be found in supplementary table 1. The number of comorbidities was significantly associated with both BASDAI and BASFI: each additional comorbidity was associated with a BASDAI increase of 0.17 points and a BASFI increase of 0.24 points independently of other factors, including treatment. Two of the most common comorbidities, depression and chronic pulmonary diseases, were associated with BASDAI increases of 0.66 and 0.38 points and BASFI increases of 0.70 and 0.34 points, respectively. Hypertension showed an independent association with BASFI in addition to depression and chronic pulmonary diseases: their presence was associated with 0.69 (complicated form) and 0.57 (uncomplicated form) point increases in the BASFI, respectively. Similar trends for depression, chronic pulmonary diseases, and hypertension were observed, although attenuated and no longer significant for chronic pulmonary diseases and complicated hypertension, when these comorbidities were only counted to be present if indicative medications were prescribed (supplementary table 2).

**Table 4:** Association of comorbidity with disease activity and functional impairment in patients with axial spondyloarthritis (*N* = 1776)

	Reference	Univariable analyses	Multivariable analyses				
			Model 1: number of comorbidities			Model 2: comorbidities taken separately	
		BASDAI, ***β*** (95% CI)	BASFI, ***β*** (95% CI)	BASDAI, ***β*** (95% CI)	BASFI, ***β*** (95% CI)	BASDAI, ***β*** (95% CI)	BASFI, ***β*** (95% CI)
Number of comorbidities	Per unit	0.23 (0.18, 0.27)	0.45 (0.40, 0.51)	0.17 (0.09, 0.24)	0.24 (0.15, 0.32)		
Depression	Not present	1.14 (0.93, 1.35)	1.12 (0.85, 1.38)			0.66 (0.41, 0.92)	0.70 (0.40, 0.99)
Chronic pulmonary disease	Not present	0.46 (0.22, 0.69)	0.79 (0.50, 1.08)			0.38 (0.10, 0.67)	0.34 (0.01, 0.66)
Hypertension (complicated)	Not present	0.41 (0.04, 0.77)	1.08 (0.60, 1.56)				0.69 (0.02, 1.35)
Hypertension (uncomplicated)	Not present	0.42 (0.23, 0.61)	1.15 (0.92, 1.39)				0.57 (0.25, 0.88)
Age	Per 10 years	0.12 (0.05, 0.18)	0.60 (0.52, 0.67)	0.06 (− 0.03, 0.16)	0.41 (0.31, 0.52)	0.14 (0.05, 0.23)	0.45 (0.34, 0.55)
Sex	Male	0.68 (0.49, 0.87)	− 0.09 (− 0.33, 0.14)	0.56 (0.33, 0.79)	− 0.01 (− 0.27, 0.26)	0.53 (0.31, 0.76)	0.00 (− 0.27, 0.26)
In rheumatologic care	No	0.46 (0.26, 0.65)	0.41 (0.17, 0.65)		0.45 (0.18, 0.73)		0.51 (0.24, 0.78)
Body mass index	Per unit	0.05 (0.03, 0.07)	0.13 (0.11, 0.16)	0.03 (0.00, 0.06)	0.08 (0.04, 0.11)	0.04 (0.01, 0.06)	0.08 (0.05, 0.11)
Smoking (current)	No	0.23 (− 0.02, 0.48)	0.17 (− 0.13, 0.46)	0.38 (0.09, 0.66)	0.58 (0.28, 0.88)	0.34 (0.06, 0.62)	0.56 (0.27, 0.86)
Suffering from stress	No	0.70 (0.51, 0.89)	− 0.28 (− 0.52, − 0.04)	0.59 (0.35, 0.82)		0.54 (0.31, 0.77)	
Lack of exercise	No	0.20 (− 0.02, 0.41)	0.85 (0.57, 1.13)		0.41 (0.10, 0.72)		0.44 (0.12, 0.75)
Household income (€)							
< 1500	> 3200	1.17 (0.89, 1.45)	1.85 (1.50, 2.20)	0.70 (0.36, 1.05)	1.11 (0.71, 1.51)	0.65 (0.30, 1.00)	1.10 (0.71, 1.50)
1500–3200	> 3200	0.72 (0.48, 0.97)	1.02 (0.73, 1.31)	0.44 (0.18, 0.71)	0.48 (0.20, 0.77)	0.41 (0.15, 0.68)	0.46 (0.17, 0.75)
NSAIDs	No	0.77 (0.57, 0.96)	0.57 (0.33, 0.82)	0.47 (0.22, 0.72)		0.43 (0.19, 0.68)	
bDMARDs	No	− 0.36 (− 0.61, − 0.11)	− 0.20 (− 0.50, 0.10)	− 0.31 (− 0.60, − 0.02)		− 0.33 (− 0.61, − 0.04)	
Non-opioid analgesics	No	0.97 (0.75, 1.19)	1.28 (1.00, 1.56)	0.46 (0.18, 0.74)	0.46 (0.13, 0.79)	0.43 (0.16, 0.71)	0.46 (0.13, 0.79)
Opioids	No	1.49 (1.25, 1.73)	2.31 (2.01, 2.61)	0.88 (0.54, 1.22)	1.41 (1.01, 1.82)	0.85 (0.52, 1.18)	1.46 (1.06, 1.86)
Steroids	No	0.68 (0.45, 0.92)	0.84 (0.54, 1.15)		0.35 (0.01, 0.68)	0.31 (0.02, 0.59)	0.39 (0.05, 0.72)
Physical therapy	No	0.71 (0.52, 0.90)	0.77 (0.53, 1.00)	0.34 (0.11, 0.58)	0.41 (0.14, 0.67)	0.35 (0.11, 0.58)	0.40 (0.14, 0.67)

Variables tested with backward selection: number of comorbidities (only model 1), comorbidities with prevalence > 5% taken separately (only model 2), age, sex, symptom duration, in rheumatologic care, HLA-B27, psoriasis, inflammatory bowel disease, uveitis, body mass index, lack of exercise, smoking, suffering from stress, household income, NSAIDs, bDMARDs, non-opioid analgesics, opioids, csDMARDs, steroids, number of pharmaceuticals (excluding axSpA-related medication), and physical therapy. Variables not included in the above table were not chosen by the backward selection in any multivariable model. Age and sex were always included in the models

*AxSpA* axial spondyloarthritis, *BASDAI* Bath Ankylosing Spondylitis Disease Activity Index, *BASFI* Bath Ankylosing Spondylitis Functional Index, *bDMARDs* biological disease-modifying anti-rheumatic drugs, *NSAIDs* non-steroidal anti-inflammatory drugs

## Discussion

The objective of this population-based study was to examine the prevalence of comorbidities and their association with disease activity and functional impairment in a large well-characterised cohort of patients with axSpA.

Our study found that comorbid conditions in axSpA are common, affecting over 80% of the cohort. This prevalence is higher than in the recent worldwide ASAS-COMOSPA cohort, where the prevalence of comorbid conditions was approximately 51% [5]. However, this is likely due to the larger number of comorbidities assessed in our study and to differences in the methodology of data collection. Hypertension was the most common comorbidity (52%) and had a higher prevalence in the current study than in the worldwide ASAS-COMOSPA cohort (34%) but was comparable to the German ASAS-COMOSPA cohort (47%). Almost all patients with complicated forms of hypertension were treated, and almost 90% of patients with uncomplicated hypertension received related pharmacological treatment. Depression was the second most prevalent comorbidity, affecting approximately a quarter of our cohort (26%), of whom about a half were treated with anti-depressants. Depression was not described in ASAS-COMOSPA; however, it has been reported elsewhere that the prevalence of depressive symptoms in axSpA patients is high [12], and depression was found to be one of the most frequent comorbidities in SpA [3]. The prevalence of diabetes, the fourth most common comorbidity, was slightly increased compared to the German ASAS-COMOSPA cohort (16% vs 12%). However, patients in our study were on average 10 years older than in the German ASAS-COMOSPA cohort and thus, that difference might be age-related.

We also examined the prevalence of comorbid conditions in non-axSpA patients matched by age and sex. The prevalence of almost all investigated comorbidities was higher in the axSpA patients than in the non-axSpA patients. Similar trends were observed in a previous study exploring the prevalence of comorbidities among Chinese patients with AS and a general Chinese population in Taiwan, which found that patients with AS have a higher prevalence of multiple comorbidities than the general population [17]. These differences are likely explained by the fact that comorbid conditions are better recognised in axSpA patients than in the general population due to the presence of a chronic disease (axSpA), which is generally associated with more physician visits and examinations. In our study, the most striking difference between patients with axSpA and non-axSpA was observed for osteoporosis (13% vs 4%). The prevalence of osteoporosis among the axSpA patients was very similar to that observed in ASAS-COMOSPA (worldwide, 13%; Germany, 15%).

Differences in disease-related characteristics across the groups of patients with none, 1–2, 3–4, and 5 or more comorbid conditions were also investigated. Patients with no comorbidities were, on average, 20 years younger, with shorter disease duration and with shorter diagnostic delay than patients with 5 or more comorbidities. This trend is comparable to what has been reported in the literature in the general population, where comorbidities increase with age [18, 19]. Furthermore, we found that patients with more comorbidities were less often in rheumatologic care and received biologic agents less often than axSpA-only patients. Similar results were demonstrated for patients with RA, showing that coverage by rheumatologic care decreases with an increasing number of comorbidities [20] and that the initiation of biological therapy was less likely in the presence of comorbidities [21, 22]. These findings are also consistent with the trend to undertreat elderly patients [23, 24] and might also be a consequence of concerns regarding adverse events or polypharmacy [25]. A striking difference in terms of lifestyle characteristics was found for suffering from stress, which was reported almost twice as much in patients with no comorbid conditions (46%) than in patients with 5 or more comorbid conditions (25%). This might be related to the proportion of patients in full-time employment, which was considerably increased among patients with no comorbid conditions (54%) compared to patients with 5 or more comorbid conditions (6%). Furthermore, patients with no comorbid conditions were middle-aged (45 years), whereas the mean age of patients with 5 or more comorbid conditions was 66 years. Thus, suffering from stress is likely related to career-oriented and family demands.

The impact of comorbidities on disease outcomes is well outlined for the general population, where the co-occurrence of at least 2 chronic diseases was associated with impaired daily functioning and lower health-related quality of life, especially if a rheumatic disease was involved [26], and for patients with RA, where increasing numbers of comorbidities were associated with worse functional status and lower well-being [20]. These associations are less well described for SpA. In separate models, analysing the impact of an increasing number of comorbidities on disease outcomes in axSpA patients, we demonstrated that the presence of each additional comorbidity was associated with a BASDAI increase of 0.17 points and a BASFI increase of 0.24 points, independently of other factors, including treatment, which is in particular relevant for patients with a high comorbidity burden. Similar results for SpA patients were found in two recent studies which showed that a rising comorbidity burden was associated with a higher disease activity, worse functional outcomes, and poorer quality of life [5, 27]. However, the difference with our study is that, in those studies, there was a selective reporting of comorbidities known to occur most commonly in SpA. When including diseases based on prevalence, important comorbidities might be overlooked and thus the proportion of axSpA patients having at least one additional comorbidity may have been affected. This potential limitation is not present in our study where comorbidities were selected using the Elixhauser coding algorithms, slightly modified by excluding rheumatoid arthritis/collagen vascular diseases and including osteoporosis and fibromyalgia. Furthermore, we demonstrated that two of the most common comorbidities, depression and chronic pulmonary disease, were associated with disease activity and functional status, highlighting the need for the careful evaluation of depressive symptoms as a part of the management strategy for axSpA. Hypertension showed an association with functional status only.

There are some limitations to our study. First, the cross-sectional design of this study does not allow conclusions to be drawn on the direction of causation. Higher disease activity and higher levels of functional disability might be indicators of more severe disease resulting in the development of comorbid conditions. In addition, axSpA patients might have been screened for comorbidities related to the disease, leading to an overestimation of the prevalence of these comorbidities and, thus, to an overestimation of the difference in the prevalence of comorbidities between axSpA patients and non-axSpA patients. Left censoring also represents a potential limitation: patients with a severe comorbidity burden might have been unable to respond to the survey or were no longer alive, preventing them from being included in the present study. However, the sample of axSpA patients was stratified by age, and thus, the error due to underestimation of the prevalence of some comorbidities is likely to be limited. Lastly, care should be taken when interpreting recorded diagnoses based on claims data that are normally collected for administrative purposes rather than for scientific purposes. The health insurance claims approach to define comorbidities enables a comprehensive assessment of comorbidities without pre-selection of particular conditions, but the diagnoses cannot be validated with this research approach. However, comorbidity-relevant drug prescriptions were examined, and furthermore, the initial diagnosis of axSpA based on claims data was validated using survey data by selecting only patients who confirmed the presence of axSpA. The characteristics of the resulting group in terms of age, sex distribution, prevalence of extra-musculoskeletal manifestations and pharmacological therapy were comparable to those of prospectively recruited axSpA cohorts [28–30]. While age, female sex, and the number of comorbidities were comparable between patients who responded to the survey and those who did not respond, there was some non-response bias regarding the prescription of axSpA-related treatment, which was increased among responders.

There are important strengths to this study, including the range and number of captured comorbidities. Comorbidities were defined using the Elixhauser coding algorithms and were not disease-specific in order to preclude the possibility of overlooking important comorbid conditions. A key strength of this study was the large population-based sample of axSpA patients and the linkage of survey data to health insurance data allowing a comprehensive assessment of comorbidities and their association with disease outcomes in a real-life setting.

In summary, we found a high prevalence of comorbidities in patients with axSpA. A higher number of comorbidities was independently associated with higher disease activity and higher levels of functional impairment while controlling for other factors, including treatment. Depression and chronic pulmonary disease were two of the most common comorbidities, and their presence was associated with higher disease activity and worse functional status, while hypertension showed an association with functional status only. These findings highlight the importance of a holistic patient-tailored management strategy in axSpA.

## Supplementary information

**Additional file 1: **A univariable association of comorbidities with disease activity and functional impairment in patients with axial spondyloarthritis (*N*=1,776). Results from univariable linear regression models analysing the association of comorbidities with a prevalence >5% with disease activity and functional impairment.

**Additional file 2:.** Association of depression, chronic pulmonary disease and hypertension with disease activity and functional impairment in a multivariable analysis in patients with axial spondyloarthritis (N=1,776). Results from multivariable linear regression models analysing the association of specific comorbidities with disease activity and functional impairment. Comorbidities were only counted to be present if indicative medications were prescribed.

## Data Availability

The data that support the findings of this study are available from the BARMER Statutory Health Insurance but restrictions apply to the availability of these data, which were used under licence for the current study, and so are not publicly available.
