# Supplementary material for: The prevalence and impact of comorbidities on patients with axial spondyloarthritis: results from a nationwide population-based study
Source: Arthritis Res Ther. 2020 Sep 10;22:210. doi: 10.1186/s13075-020-02301-0 (PMC7488243; doi:10.1186/s13075-020-02301-0)
Supplement: Supplementary file 1 — Additional file 1: A univariable association of comorbidities with disease activity and functional impairment in patients with axial spondyloarthritis (N=1,776). Results from univariable linear regression models analysing the association of comorbidities with a prevalence >5% with disease activity and functional impairment. [file 13075_2020_2301_MOESM1_ESM.pdf]

Supplementary table 1 A univariable association of comorbidities* with disease activity and functional impairment in patients with axial spondyloarthritis (N=1,776)

	**Reference**	**BASDAI**<br>**β (95% CI)**	**BASFI**<br>**β (95% CI)**
Hypertension (complicated)	not present	0.41 (0.04, 0.77)	1.08 (0.60, 1.56)
Hypertension (uncomplicated)	not present	0.42 (0.23, 0.61)	1.15 (0.92, 1.39)
Depression	not present	1.14 (0.93, 1.35)	1.12 (0.85, 1.38)
Chronic pulmonary disease	not present	0.46 (0.22, 0.69)	0.79 (0.51, 1.08)
Diabetes (complicated)	not present	0.47 (0.12, 0.82)	1.32 (0.90, 1.75)
Diabetes (uncomplicated)	not present	0.25 (-0.13, 0.62)	0.92 (0.46, 1.38)
Cardiac arrhythmias	not present	0.23 (-0.05, 0.51)	0.79 (0.44, 1.15)
Obesity	not present	0.61 (0.34, 0.88)	1.37 (1.04, 1.71)
Hypothyroidism	not present	0.31 (0.03, 0.59)	0.35 (0.00, 0.71)
Osteoporosis	not present	0.14 (-0.16, 0.44)	0.78 (0.44, 1.13)
Liver disease	not present	0.52 (0.23, 0.81)	0.59 (0.23, 0.95)
Peripheral vascular disorders	not present	0.39 (0.04, 0.73)	1.29 (0.85, 1.74)
Solid tumor without metastasis	not present	-0.09 (-0.41, 0.24)	0.63 (0.21, 1.05)
Valvular disease	not present	0.42 (0.08, 0.76)	0.39 (-0.03, 0.81)
Renal failure	not present	0.26 (-0.11, 0.64)	1.09 (0.63, 1.56)
Congestive heart failure	not present	0.49 (0.09, 0.89)	1.83 (1.34, 2.33)
Deficiency anemia	not present	0.28 (-0.16, 0.73)	0.37 (-0.20, 0.94)

* with prevalence >5%.

BASDAI, Bath Ankylosing Spondylitis Disease Activity Index; BASFI, Bath Ankylosing Spondylitis Functional Index.
