# Supplementary material for: The prevalence and impact of comorbidities on patients with axial spondyloarthritis: results from a nationwide population-based study
Source: Arthritis Res Ther. 2020 Sep 10;22:210. doi: 10.1186/s13075-020-02301-0 (PMC7488243; doi:10.1186/s13075-020-02301-0)
Supplement: Supplementary file 2 — Additional file 2:. Association of depression, chronic pulmonary disease and hypertension with disease activity and functional impairment in a multivariable analysis in patients with axial spondyloarthritis (N=1,776). Results from multivariable linear regression models analysing the association of specific comorbidities with disease activity and functional impairment. Comorbidities were only counted to be present if indicative medications were prescribed. [file 13075_2020_2301_MOESM2_ESM.pdf]

Supplementary table 2 Association of depression, chronic pulmonary disease and hypertension* with disease activity and functional impairment in a multivariable analysis in patients with axial spondyloarthritis (N=1,776)

	**Reference**	**BASDAI** **β (95% CI)**	**BASFI** **β (95% CI)**
Depression	not present	0.52 (0.26, 0.79)	0.59 (0.27, 0.92)
Chronic pulmonary disease	not present	0.21 (-0.10, 0.52)	0.26 (-0.11, 0.62)
Hypertension (complicated)	not present		0.40 (-0.11, 0.90)
Hypertension (uncomplicated)	not present		0.38 (0.11, 0.65)
Age	per 10 years	0.14 (0.06, 0.21)	0.50 (0.41, 0.59)
Sex	male	0.55 (0.37, 0.74)	0.00 (-0.22, 0.22)
In rheumatologic care	no		0.61 (0.38, 0.84)
Body mass index	per unit	0.03 (0.01, 0.05)	0.07 (0.05, 0.10)
Smoking (current)	no	0.28 (0.03, 0.52)	0.52 (0.26, 0.78)
Suffering from stress	no	0.73 (0.54, 0.93)	
Lack of exercise	no		0.62 (0.36, 0.87)
Household income (€)			
<1500	>3200	0.80 (0.53, 1.08)	1.08 (0.76, 1.40)
1500-3200	>3200	0.53 (0.29, 0.76)	0.49 (0.24, 0.75)
NSAIDs	no	0.47 (0.27, 0.66)	
bDMARDs	no	-0.28 (-0.54, -0.02)	
Non-opioid analgesics	no	0.37 (0.15, 0.60)	0.43 (0.16, 0.71)
Opioids	no	0.99 (0.73, 1.26)	1.63 (1.30, 1.95)
Steroids	no	0.33 (0.09, 0.56)	0.26 (-0.01, 0.54)
Physical therapy	no	0.35 (0.17, 0.54)	0.33 (0.12, 0.55)

*All comorbidities were only assumed to be present if indicative medications were prescribed.

BASDAI, Bath Ankylosing Spondylitis Disease Activity Index; BASFI, Bath Ankylosing Spondylitis Functional Index; bDMARDs, biological disease-modifying anti-rheumatic drugs; NSAIDs, non-steroidal anti-inflammatory drugs.
